# Secreted Osteopontin Is Highly Polymerized in Human Airways and Fragmented in Asthmatic Airway Secretions

**DOI:** 10.1371/journal.pone.0025678

**Published:** 2011-10-21

**Authors:** Mehrdad Arjomandi, Jessica Frelinger, Aneesh Donde, Hofer Wong, Amritha Yellamilli, Wilfred Raymond

**Affiliations:** 1 Division of Pulmonary and Critical Care Medicine, Department of Medicine, University of California San Francisco, San Francisco, California, United States of America; 2 Human Exposure Laboratory, Department of Medicine, University of California San Francisco, San Francisco, California, United States of America; 3 Cardiovascular Research Institute, Department of Medicine, University of California San Francisco, San Francisco, California, United States of America; 4 Pulmonary Research Group, San Francisco Veterans Affairs Medical Center, San Francisco, California, United States of America; University of Leuven, Rega Institute, Belgium

## Abstract

**Background:**

Osteopontin (OPN) is a member of the small integrin-binding ligand N-linked glycoprotein (SIBLING) family and a cytokine with diverse biologic roles. OPN undergoes extensive post-translational modifications, including polymerization and proteolytic fragmentation, which alters its biologic activity. Recent studies suggest that OPN may contribute to the pathogenesis of asthma.

**Methodology:**

To determine whether secreted OPN (sOPN) is polymerized in human airways and whether it is qualitatively different in asthma, we used immunoblotting to examine sOPN in bronchoalveolar lavage (BAL) fluid samples from 12 healthy and 21 asthmatic subjects (and in sputum samples from 27 healthy and 21 asthmatic subjects). All asthmatic subjects had mild to moderate asthma and abstained from corticosteroids during the study. Furthermore, we examined the relationship between airway sOPN and cellular inflammation.

**Principal Findings:**

We found that sOPN in BAL fluid and sputum exists in polymeric, monomeric, and cleaved forms, with most of it in polymeric form. Compared to healthy subjects, asthmatic subjects had proportionately less polymeric sOPN and more monomeric and cleaved sOPN. Polymeric sOPN in BAL fluid was associated with increased alveolar macrophage counts in airways in all subjects.

**Conclusions:**

These results suggest that sOPN in human airways (1) undergoes extensive post-translational modification by polymerization and proteolytic fragmentation, (2) is more fragmented and less polymerized in subjects with mild to moderate asthma, and (3) may contribute to recruitment or survival of alveolar macrophages.

## Introduction

Osteopontin (OPN) is a small integrin-binding ligand N-linked glycoprotein (SIBLING) and a cytokine with diverse roles in tissue remodeling, fibrosis, immunomodulation, inflammation, and tumor metastasis [1], [2], [3], [4], [5], [6], [7]. It is synthesized at the highest levels in bone and epithelial tissues [8], [9], but is also made by activated T lymphocytes [10], [11], smooth muscle cells [12], and cells associated with the reticuloendothelial system, including macrophages and dendritic cells [13], [14], [15]. While initially thought to be exclusively a secreted protein (sOPN), later evidence showed that OPN also possesses intracellular forms (iOPN) [16], [17].

Osteopontin undergoes major post-translational modification, including phosphorylation at up to 36 sites and N- and O-linked glycosylation, with subsequent increase in molecular weight of nascent protein from 34 kD to 44–65 kD post-modification [8], [18]. It contains an Arg-Gly-Asp (RGD) motif that mediates cell attachment and signaling via its reaction with cell-surface integrins [19]. Osteopontin is also a target of proteolytic cleavage by proteases such as thrombin, plasmin, and cathepsin D, which exposes a cryptic sequence (SVVYGLR) that binds to several integrin receptors through which it mediates recruitment of inflammatory cells including neutrophils and macrophages [20], [21], [22], [23], [24], [25], [26]. Furthermore, OPN is a substrate for tissue transglutaminase (TGM2) and can undergo polymerization (as well as cross-linkage to fibronectin) via transglutamination at Glu^50^-Lys^51^-Glu^52^ residues near its N-terminus, which generates novel integrin binding sites [8], [27], [28], [29], [30], [31], [32]. These post-translational modifications change structure, affect biological properties, and may explain the diversity of roles that sOPN plays in physiologic and pathologic processes [27], [32].

Recent evidence suggests that OPN may play a role in the pathogenesis of asthma. Several investigators have shown that OPN influences the pathophysiology of murine models of allergic airway disease. Osteopontin deficiency, generated by administration of blocking antibody or by genomic alteration (knockout mice), is reported to protect against airway hyperresponsiveness (AHR) and airway remodeling [33], [34], [35], [36], [37]. The state of polymerization or fragmentation of OPN in these mice has not been determined. In humans, polymorphism in the OPN gene (SPP1) has been shown to be associated with asthma in a Puerto Rican population [38]. Immunohistochemical analysis of endobronchial biopsies has shown increased OPN expression in bronchial epithelium and subepithelial inflammatory cells in asthmatic subjects compared to non-asthmatic controls [33]. In addition, four recent studies using ELISA assays revealed increased levels of sOPN in BAL fluid and induced sputum in asthmatic subjects [39], [40], [41], [42]. However, the conformation of sOPN in airways has not been characterized, nor has it been shown whether there are differences in post-translational modification of sOPN between healthy and asthmatic subjects, particularly as TGM2, which polymerizes sOPN, is reported to be more abundant in certain asthmatic subjects [43].

Given the above observations, we hypothesized (1) that sOPN exists in different conformations including polymeric forms in human airways, and (2) that the reported difference between healthy and asthmatic subjects in expression of OPN is due to higher abundance of polymeric sOPN in asthmatic airways. To test these hypotheses, we characterized sOPN in human respiratory tract lining fluid (RTLF, as represented by BAL and induced sputum) from healthy and asthmatic subjects.

## Methods

All work performed in this research was approved by the University of California institutional review board, the Committee on Human Research (CHR). Subjects were informed of the risks of the experimental protocol and signed a CHR-approved consent form. All subjects received financial compensation for their participation.

### Study design

This study had a cross-sectional observational design and involved two visits. During the first visit, subjects were consented and then underwent history and physical examination, completion of a medical questionnaire, spirometry, skin prick testing, and methacholine challenge testing. Subjects were then assigned to sputum induction or bronchoscopy groups. During the second visit, subjects underwent spirometry with and without bronchodilator, and sputum induction or bronchoscopy according to their group assignment.

### Human subjects

Please see Methods S1 for full details of human subject, bronchoscopy, and sputum induction methods.

Subjects were recruited by public advertisements, and were included in the study if they had a history of physician-diagnosed asthma and AHR to inhaled methacholine (provocative concentration of methacholine resulting in a 20% decrease in FEV_1_ compared with post-diluent [PC_20_] ≤8.0 mg/ml), verified in our laboratory according to a standard protocol [44]. Healthy non-asthmatic control subjects also were included in the study if they had no history of asthma and had a negative methacholine challenge test as defined by [PC_20_] >16.0 mg/ml.

Subjects were excluded if they had a history of cardiac or pulmonary diseases other than asthma, oral corticosteroid use within the 3 months prior to enrollment, or respiratory infection within 6 weeks prior to enrollment. Subjects were excluded if they had more than 1 pack-year lifetime history of prior tobacco use or had smoked within 6 months of participating in the study, or if they had a history of intravenous or inhaled illicit drug use. To avoid any possible corticosteroid effects, subjects were requested to withhold inhaled corticosteroids for at least 2 weeks before each study session. Short-acting inhaled bronchodilators were allowed throughout the course of the study.

### *In vitro* polymerization of rOPN by transglutaminase 2

**Figure 1:**
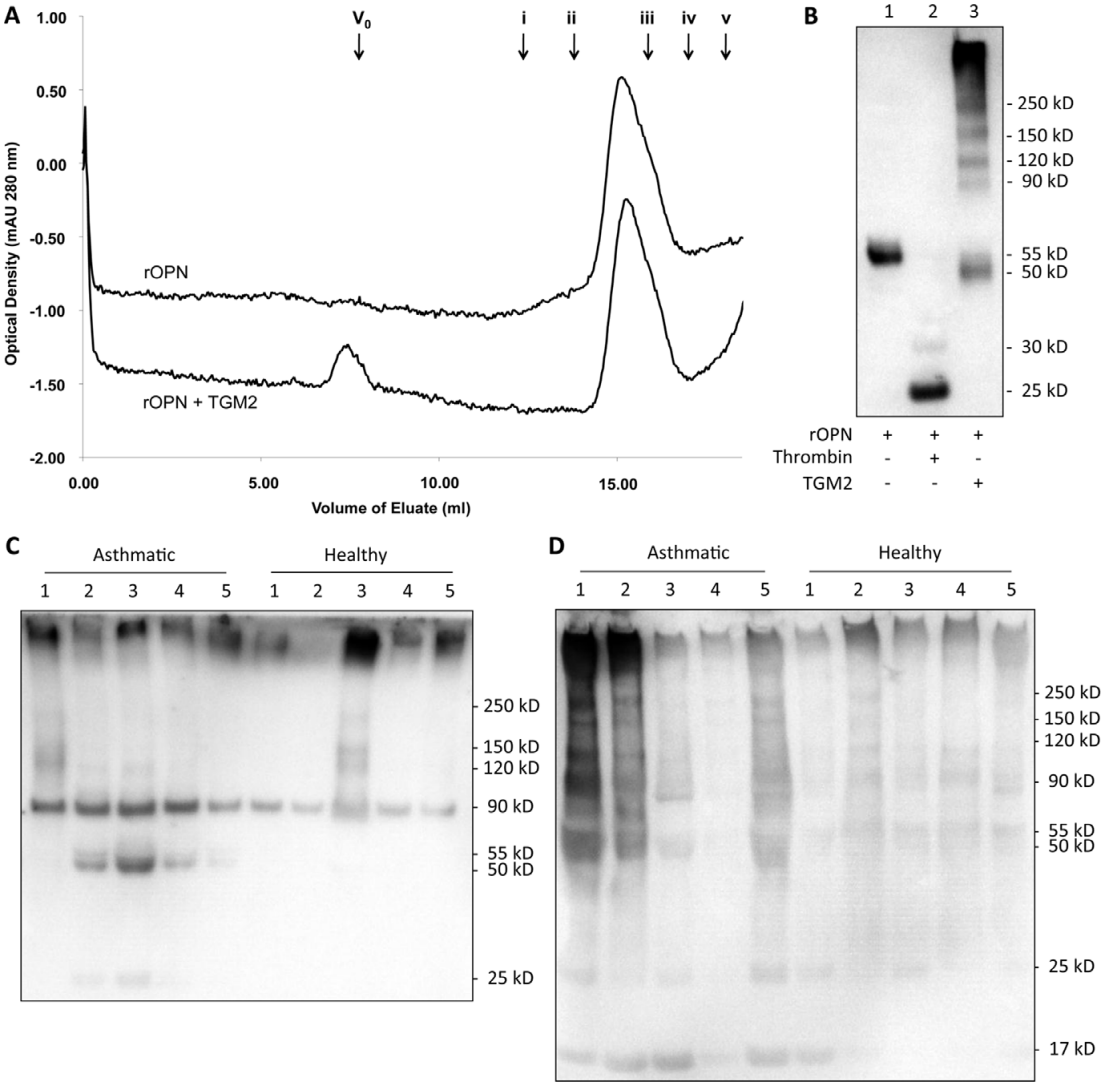
Monomeric, cleaved, and polymeric rOPN, BAL, and sputum sOPN. **1A-** Size exclusion chromatogram of rOPN and TGM2-treated (polymerized) rOPN; V_0_: void volume; i: thyroglobulin (669 kD); ii: apoferritin (460 kD); iii: gamma globulin (158 kD); iv: bovine serum albumin (65.5 kD); v: human chymase (30 kD). **1B-** Immunoblot of rOPN, thrombin-cleaved rOPN, and TGM2-treated (polymerized) rOPN. Lane 1: rOPN; Lane 2: rOPN treated with thrombin; Lane 3: polymeric rOPN made using TGM2. **1C-** Immunoblot of BAL samples from 5 healthy and 5 asthmatic subjects probed with affinity purified polyclonal anti-human sOPN antibody. **1D-** Immunoblot of induced sputum samples from 5 healthy and 5 asthmatic subjects probed with affinity purified polyclonal anti-human sOPN antibody.

Human rOPN (1 µg/ml) (1433-OP/CF; R&D Systems; Minneapolis, MN) was incubated with recombinant human transglutaminase 2 (TGM2) (0.06 µg/ml) (4376-TG-050; R&D Systems) in reaction buffer consisting of 5 mM CaCl_2_, 1 mM DTT, and 50 mM Tris-HCl (pH 7.5) at 37°C for 2 h (Figure 1B).

### *In vitro* digestion of rOPN by thrombin

Human rOPN (1 µg/ml) or TGM2-treated (polymeric) rOPN (1 µg/ml) was incubated with recombinant human thrombin (0.02 µg/ml) (1473-SE; R&D Systems) in reaction buffer (0.1 M ammonium bicarbonate) or in PBS at 37°C for 2 h (Figure 1B).

### *In vitro* dephosphorylation and deglycosylation of rOPN and sOPN

Samples were deglycosylated using a Protein Deglycosylation Mix kit (P6039; New England Biolabs Inc., Ipswich, MA). Human rOPN or human BAL (sOPN) was added to 10x glycoprotein Denaturing Buffer, denatured by heating at 100°C for 10 min and then incubated with 10x G7 Reaction Buffer and Deglycosylation Enzyme Cocktail at 37°C for 4 h according to manufacturer protocol (New England Biolabs). Human rOPN and BAL (sOPN) were dephosphorylated with alkaline phosphatase (M0290S; New England Biolabs) at 37°C for 2 h.

### Western blot materials

Goat affinity-purified anti-human OPN polyclonal antibody (AF1433) raised against natural human milk sOPN, anti-goat IgG-HRP antibody (AB-108-C), and human rOPN (1433-OP) made in a murine myeloma cell line were purchased from R&D Systems. BSA, Tris-HCl, and Tween 20 were obtained from Sigma-Aldrich (St. Louis, MO). Polymeric and thrombin-cleaved rOPN were synthesized as described above.

### Denaturing Western blot

Immunoblotting was performed using Invitrogen XCell SureLock Mini-Cell and XCell II BlotModule kit and reagents and NuPAGE 4–12% gradient Bis-Tris gels (Life Technologies, Carlsbad, CA). A total of 10 µl of each BAL or sputum fluid samples was loaded on the gel. Protein concentrations were measured separately and the optical densities were adjusted for protein concentration as detailed below. Control recombinant proteins (rOPN, polymeric and cleaved rOPN) as well as BAL and sputum samples were reduced and denatured with 5 mM dithiothreitol at 90°C for 10 min, electrophoresed by SDS-PAGE, and transferred to polyvinylidene difluoride membranes (Invitrogen, Life Technologies, Carlsbad, CA) for immunodetection. The membranes were blocked in 2% Tween in Tris-buffered saline (TBS) containing BSA and probed using anti-human OPN polyclonal antibodies overnight at 4°C and then detected using appropriate horseradish peroxidase (HRP)-conjugated antibodies and West-Dura chemiluminescent substrate. The chemiluminescent signal was visualized using Fluor Chem FC2 System (Cell Biosciences, Santa Clara, CA).

### Optical densitometry

Optical density was measured using ImageJ software (version1.44, NIH, Bethesda, MD). Each sOPN band was measured separately. The polymeric sOPN bands that produced a continuous smear were measured as a single band. Other polymeric bands were measured separately. For the purpose of analysis, consistent with the described molecular weights of various forms of sOPN and with our polymerization and cleavage experiment, bands from 40 up to 90 kD were considered to be monomeric. Bands ≥90 kD were considered as polymeric (bands between 90 and 100 kD were considered dimeric sOPN) and bands <40 kD were considered to be cleaved.

For each subject, relative densitometry was determined via measurement of the signal from each sOPN moiety relative to the cumulative sOPN bands signal [for example, monomeric sOPN relative OD = OD_monomeric sOPN_/(OD_polymeric sOPN_+OD_monomeric sOPN_+OD_cleaved sOPN_].

Absolute densitometry for each band was determined as relative signal intensity to the appropriate size rOPN control band signal [for example, monomeric sOPN densitometry unit = OD_monomeric sOPN_/OD_monomeric rOPN 20 ng control_ and polymeric sOPN densitometry unit = OD_polymeric sOPN_/OD_polymeric rOPN 20 ng control_]. The signal intensity of polymeric sOPN was compared to the signal intensity of smear band of 20 ng of synthesized polymeric rOPN.

### Size exclusion (gel filtration) chromatography

Human rOPN samples [rOPN or TGM2-treated (polymeric) rOPN] were injected onto a Superose 6 10/300 GL column (GE Healthcare Life Sciences, Piscataway, NJ) equilibrated with phosphate buffered saline (PBS, pH 7.4). The column was calibrated on separate runs with thyroglobulin (669 kD), apoferritin (460 kD), gamma globulin (158 kD), bovine serum albumin (65.5 kD), and human chymase (30 kD).

### Sample size and power calculations

Previous human studies reported detecting significant differences in ELISA-measured OPN concentrations with sample sizes of 17 to 35 subjects in each healthy or asthmatic group [39], [40], [41], [45]. We based our sample size on the ability to detect a 10% difference in optical density of sOPN moieties. A sample size of 35 for BAL fluid samples provided statistical power of 81% with a two-sided type I error of 5% to observe a minimal difference of 10% with the assumption that the true difference between optical densities of sOPN moieties is the same as its standard deviation; our sample of 33 subjects provided a power of 79%. For sputum samples, given the reported larger variance in endpoints measured in sputum versus BAL [46], we assumed the true difference between healthy and asthmatic subjects to be 0.85 of its standard deviation. A sample size of 50 thus provided statistical power of 83% with a two-sided type I error of 5% to observe a minimal difference of 10%; our sample of 47 subjects provided a power of 81%.

### Data management and analysis

All data were entered into a database developed in Microsoft Excel 2000 (Microsoft, Redmond, WA). The absolute optical densities from immunoblots were adjusted for protein concentration of corresponding BAL or sputum samples by division of the signal by protein concentration or were adjusted in multivariate regression models. The relative optical densities were not adjusted as they were measured and calculated within the same sample from the same subject.

Data were analyzed using STATA 10.0 software (STATA Corp, College Station, TX). Student's *t*-test (parametric data) and Mann–Whitney *U* test (non-parametric data) were used for comparisons between asthmatic and non-asthmatic subjects. P-values less than 0.05 were considered to be statistically significant.

## Results

### Study subjects, BAL, and induced sputum

BAL fluid samples were obtained from 33 subjects (12 healthy and 21 asthmatic subjects, Table S1). Induced sputum samples were obtained from 47 subjects (27 healthy and 21 asthmatic subjects, Table S2). All asthmatic subjects who were taking inhaled corticosteroid discontinued their use 2 weeks prior to collection of BAL or sputum. The concentrations of inflammatory cells in BAL and sputum samples are shown in Tables S3 and S4. The concentration of total protein in BAL samples was not significantly different between healthy and asthmatic subjects.

### Secreted OPN exists in polymeric, monomeric, and cleaved forms in BAL and sputum

Immunoblotting of BAL samples with affinity-purified anti-human OPN polyclonal antibody showed several different forms of sOPN: a smeared band >250 kD along with a few other bands in the 100 to 250 kD range representing polymeric (multimeric) sOPN, a band at 90 kD likely representing dimeric sOPN, two bands at 50 and 55 kD representing monomeric sOPN, and a band at 25 kD representing cleaved sOPN (Figure 1C). The molecular weights of these bands correlated with those of our synthetic polymeric (TGM2-treated), monomeric, and thrombin-cleaved rOPN bands (Figure 1B). The sputum samples showed a similar pattern of bands as well as an additional band at 17 kD likely representing an additional (non-thrombin) cleaved sOPN (Figure 1D). To demonstrate polymerization of rOPN by TGM2 through an alternate technique, size fractionation of rOPN (monomeric) or TGM2-treated rOPN (polymeric) was performed using a Superose 6 gel filtration column equilibrated with PBS at pH 7.4. Size fractionation showed that rOPN (monomeric rOPN that migrated at a molecular weight of 55 kD on SDS-PAGE) eluted at a single peak in the 200–240 kD range (Figure 1A), suggesting that in phosphate buffered saline (PBS) and physiologic pH, rOPN behaves as an oligomer (possibly tetramer). In contrast, TGM2-treated rOPN (polymeric) eluted at two peaks: one in the void volume (>5000 kD) and another identical to untreated rOPN in the 200–240 kD range (Figure 1A), suggesting that the TGM2-treated rOPN produced a highly polymerized large moiety that eluted with the void volume.

### Secreted OPN is highly glycosylated and phosphorylated in BAL and sputum

**Figure 2:**
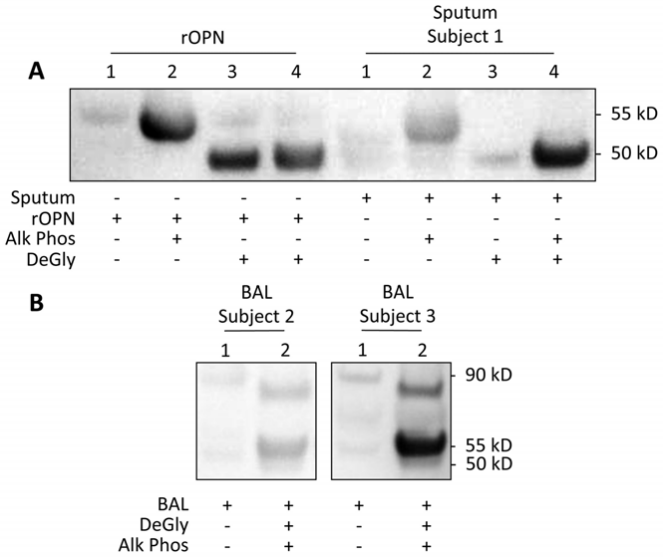
Deglycosylation and dephosphorylation of rOPN and BAL and sputum sOPN. Immunoblot of deglycosylation and dephosphorylation of rOPN and sputum supernatant sOPN (**2A**) and BAL fluid sOPN from representative subjects (**2B**) probed with polyclonal anti-human sOPN antibody. DeGly: Deglycosylation Enzyme Mix; Alk Phos: Alkaline Phosphatase.

Deglycosylation and dephosphorylation of rOPN decreased the size of the rOPN band from 55 kD to 50 kD (Figure 2A). Similarly, treatment of BAL samples showed the appearance of a second band of sOPN at 50 kD (Figure 2B). In addition, the deglycosylation and dephosphorylation caused an analogous shift in the 90 kD band of sOPN down to about 80 kD. No significant changes were observed in the polymeric sOPN band in BAL. The sputum samples showed a similar pattern of shift in their bands (Figure 2A).

### Most sOPN in BAL and sputum is in polymeric form

**Figure 3:**
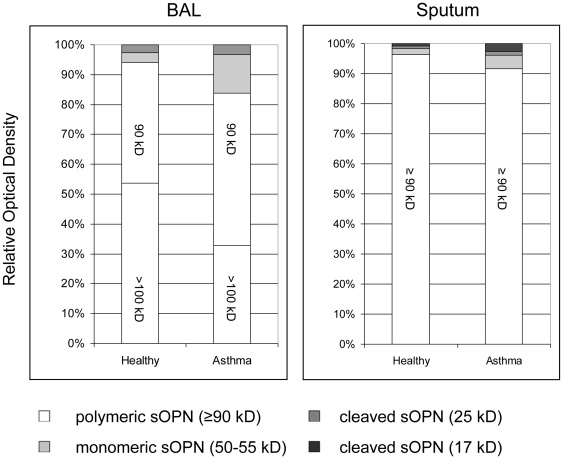
Secreted OPN in BAL and sputum. Relative optical density of polymeric, monomeric, and cleaved sOPN in BAL fluid and induced sputum of healthy and asthmatic subjects. White: polymeric sOPN (≥90 kD); light gray: monomeric sOPN (50–55 kD); dark gray: cleaved sOPN (25 kD); black: cleaved sOPN (17 kD).

Relative densitometry of the individual BAL and sputum samples from each subject showed that the intensity of the polymeric sOPN bands was much higher relative to the intensity of the other forms of sOPN, suggesting that most of sOPN in BAL and sputum is in polymeric form (Figure 3). The polymeric sOPN OD accounted for 86.9±13.1% and 94.2±5.8% in BAL and sputum in all subjects.

### Asthmatic BAL and sputum samples have lower fraction of polymeric and higher fraction of monomeric and cleaved sOPN

**Figure 4:**
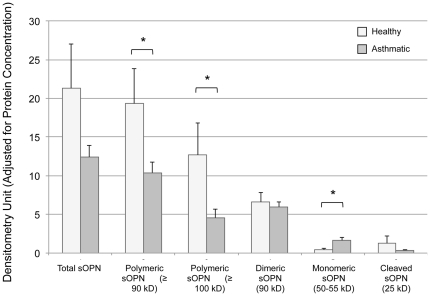
Densitometry of sOPN in BAL. Absolute optical density adjusted for protein content of polymeric, monomeric, and cleaved sOPN in BAL fluid of healthy and asthmatic subjects.

Relative densitometry showed that compared to healthy subjects, asthmatic subjects had a proportionately lower fraction of polymeric sOPN and a higher fraction of monomeric sOPN in BAL and sputum samples (Tables 1 and 2). In addition, asthmatic subjects had a higher fraction of the 17 kD cleaved sOPN seen in the sputum samples (Table 2). Similarly, in BAL samples, absolute densitometry adjusted for protein concentration also showed that the OD of polymeric sOPN was significantly higher and the OD of monomeric sOPN was significantly lower in healthy subjects compared to that of asthmatic subjects (Figure 4). In induced sputum, absolute densitometry adjusted for protein concentration showed a higher fraction of the 17 kD cleaved sOPN band in asthmatic subjects (0.2±0.2 versus 0.1±0.1 densitometry unit/mg protein in asthmatic versus healthy subjects, respectively, Mann-Whitney p = 0.036), but no statistically significant difference was detected in other bands.

**Table 1:** Relative optical density (OD) for sOPN bands and OD-based concentration of sOPN in BAL.

BAL	sOPN (µg/ml)	Relative OD	Relative OD	Relative OD	p-value
	All subjects (N = 33)	All subjects (N = 33)	Non-asthmatic Subjects (N = 12)	Asthmatic Subjects (N = 21)	
**Polymeric sOPN (90 & >100 kD)**	1.32±1.29	86.9±13.1	94.1±9.0	83.8±13.5	**0.019**
	0.99 [0.62, 1.57]	90.1 [77.9, 100.0]	100.0 [85.3, 100.0]	81.1 [76.2, 95.7]	
**Polymeric sOPN (>100 kD)**	0.78±1.16	39.1±27.0	53.7±25.2	32.8±25.7	**0.048**
	0.45 [0.07, 0.73]	34.6 [13.6, 61.4]	61.4 [35.6, 68.7]	28.4 [8.7, 51.0]	
**Dimeric sOPN (90 kD)**	0.55±0.32	47.9±25.1	40.3±23.7	51.1±25.6	0.248
	0.47 [0.25, 0.75]	44.8 [29.9, 66.7]	38.6 [22.0, 49.7]	47.6 [31.5, 67.2]	
**Monomeric sOPN (50–55 kD)**	0.15±0.19	9.9±10.3	3.2±5.3	12.8±10.7	**0.008**
	0.11 [0.00, 0.21]	6.6 [0.0, 17.0]	0.0 [0.0, 5.7]	15.0 [4.3, 18.2]	
**Cleaved sOPN (25 kD)**	0.06±0.16	3.2±4.3	2.7±5.5	3.3±0.8	0.199
	0.02 [0.00, 0.05]	1.1 [0.0, 4.6]	0.0 [0.0, 0.0]	3.1 [0.0, 4.6]	

Values are expressed as mean ± SD and median [interquartile range]. Total concentration of sOPN were 1.54±1.43 (1.17 [0.80, 1.78] in BAL. P-values are for comparisons (Mann-Whitney test) of relative OD of various bands between healthy and asthmatic subjects. Significant p-values are shown in bold.

**Table 2:** Relative optical density (OD) for sOPN bands and OD-based concentration of sOPN in sputum.

Sputum	sOPN (µg/ml)	Relative OD	Relative OD	Relative OD	p-value
	All subjects (N = 33)	All subjects (N = 47)	Non-asthmatic Subjects (N = 27)	Asthmatic Subjects (N = 20)	
**Polymeric sOPN (≥90 kD) (%)**	2.45±2.97	94.2±5.8	96.4±3.2	91.6±1.6	**0.009**
	1.59 [0.58, 2.67]	94.6 [92.7, 98.7]	96.5 [94.6, 99.4]	94.0 [89.0, 97.5]	
**Monomeric sOPN (50–55 kD) (%)**	0.28±0.47	3.2±3.3	2.0±1.8	4.5±4.1	**0.025**
	0.08 [0.00, 0.37]	2.6 [0.7, 4.4]	1.9 [0.0, 2.9]	3.8 [1.6, 6.2]	
**Cleaved sOPN (25 kD) (%)**	0.10±0.15	1.0±1.3	0.7±1.1	1.4±1.5	0.064
	0.08 [0.00, 0.08]	0.5 [0.0, 1.5]	0.3 [0.0, 0.9]	1.2 [0.3, 1.6]	
**Cleaved sOPN (17 kD) (%)**	0.11±0.17	1.7±1.9	0.9±1.4	2.6±2.0	**0.005**
	0.03 [0.01, 0.13]	0.9 [0.3, 2.7]	0.6 [0.0, 1.0]	2.7 [0.6, 4.1]	

Values are expressed as mean ± SD and median [interquartile range]. Total concentration of sOPN was 3.03±3.41(2.25 [0.80, 3.08]) µg/ml in sputum samples. P-values are for comparisons (Mann-Whitney test) of relative OD of various bands between healthy and asthmatic subjects. Significant p-values are shown in bold.

### Polymeric sOPN is associated with concentration of alveolar macrophages in BAL in all subjects

**Figure 5:**
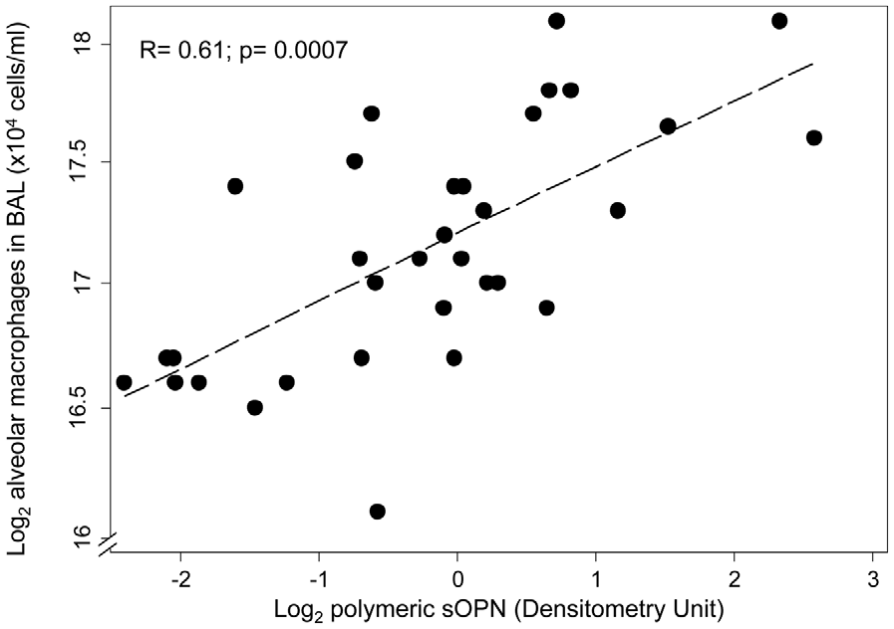
Alveolar macrophages and sOPN. Correlation between alveolar macrophage concentration (log base 2) and polymeric sOPN optical density (log base 2) in BAL (R = 0.61; p = 0.0007).

The inflammatory cell counts in BAL samples of subjects are shown in Table S2. In linear regression models, BAL polymeric sOPN concentration (as measured by absolute optical density) showed a significant association with increased cellularity in BAL (correlation coefficient R = 0.66; p = 0.0002) and in particular with alveolar macrophage concentration in BAL (Figure 5) (correlation coefficient R = 0.61; p = 0.0007). These models suggest that for every doubling concentration of polymeric sOPN in BAL, the leukocyte count in BAL increased by a factor of 24.0% (95% CI: 12.0% to 37.3%), and alveolar macrophage count in BAL increased by a factor of 22.1% (95% CI: 9.7% to 35.9%). Inclusion of age, sex, BMI, or BAL total protein concentration did not significantly affect the model. There was no association between inflammatory cells and monomeric or cleaved sOPN in BAL samples. The concentration of sOPN in sputum measured by densitometry did not correlate with sputum cell count.

## Discussion

In this study, we found that (1) human RTLF contains sOPN in polymeric, monomeric, and cleaved forms; (2) most of sOPN in RTLF is in polymeric form; and (3) polymeric sOPN is associated with higher cellular inflammation, and in particular higher concentration of alveolar macrophages, in RTLF. Furthermore, we found that monomeric rOPN behaves as a non-covalently-bound oligomer under physiologic condition. While polymeric sOPN has been identified in bone and calcified aorta [47], [48] and the presence of cleaved sOPN fragments has been detected in the synovial fluid of patients with rheumatoid arthritis [49], our study is the first to report the presence of polymeric and cleaved sOPN in airways. In addition, we report that the sOPN in human RTLF is highly glycosylated and phosphorylated. Finally, we found that, contrary to our hypothesis, compared to healthy non-asthmatic subjects, asthmatic subjects had a lower fraction of polymeric and a higher fraction of monomeric and cleaved sOPN in RTLF.

Our finding that most of sOPN exists in polymeric form is important since it has been demonstrated that post-translational modifications of OPN (such as proteolytic cleavage, polymerization, phosphorylation, and glycosylation) can significantly change its structure and biological function. Studies have shown that OPN is capable of polymerization either with itself or with other extracellular matrix proteins such as fibronectin through the catalytic action of tissue transglutaminase 2 (TGM2) [28], [31]. The elution pattern on size fractionation along with SDS-PAGE gel migration behavior of polymeric rOPN in our study suggests that polymeric sOPN in human airways also forms a large covalently-bound structure with molecular weight >5000 kD. While the functional relevance of this enormous molecule may be evident in bone tissue, its functional role in human airways is unclear and demands further investigation. Polymeric OPN has been reported to increase its binding to collagen, and enhance its biologic activity including increased integrin binding, which results in enhanced cell adhesion, spreading, migration, and focal contact formation [27], [50], [51]. Recent *in vitro* and animal studies have shown that polymerization of OPN causes a gain of chemotactic activity for neutrophils through its interaction with α9β1 integrin [27], [32]. However, further studies are needed to determine polymeric sOPN function in human airways. In addition, our finding that the rOPN behaves as a non-covalently-bound oligomer *in vitro* under physiologic condition is interesting and needs further investigation to determine whether sOPN *in vivo* also behaves as an oligomer.

Previous studies also have shown that OPN has a conserved thrombin cleavage site. Thrombin cleavage exposes a new carboxyl-terminus (SVVYGLR) in the cleaved OPN (known as OPN-R) that interacts with several other integrins in a non-RGD-dependent manner [52], [53], [54], [55], [56]. The role of SVVYGLR in thrombin-cleaved OPN has been recently demonstrated by reports that in a murine model of rheumatoid arthritis a monoclonal antibody directed against this site inhibits proliferation of synovium, infiltration of inflammatory cells, and development of bone erosions. [57] In addition to thrombin, other proteases such as plasmin and cathepsin D were reported to be able to cleave OPN to produce fragments of smaller size (14 to 19 kD) by plasmin and cathepsin-D [58]. In our study, we found that a significant portion of non-polymeric sOPN in sputum is in a cleaved form (17 kD) smaller than the thrombin-cleaved rOPN. The presence of this band, which was absent in BAL samples, may be explained by alternate phosphorylation/glycosylation sites, cleavage by alternate protease(s) such as plasmin or cathepsin D, and/or alternate sources of sOPN production within the upper airways or saliva that are absent in lower airways. Further studies are needed to determine the structure of the observed RTLF sOPN fragments, proteases involved in their formation, and their functional consequence.

Furthermore, we found a significant association between polymeric sOPN and concentration of alveolar macrophages in BAL. These findings suggests a chemotactic and/or anti-apoptotic role of polymeric sOPN for alveolar macrophages in airways, and are consistent with other studies showing that OPN acts as a direct chemoattractant and as a survival factor for inflammatory cells such as macrophages [25], [27], [59], [60], [61], [62], [63], [64], [65]. While polymeric OPN was reported to have enhanced neutrophil chemotactic ability compared to monomeric OPN [27], [32], we did not observe a relationship between polymeric sOPN and concentration of neutrophils in BAL. However, in the absence of a stimulus for neutrophilia such as airway injury, the concentration of neutrophils in BAL is very low (about 3% of total leukocyte count), and thus the presence of an association may be difficult to show with such low fractions of neutrophils. Further studies of polymeric sOPN in models of airway injury such as oxidative injury, smoke injury, infection, or acute respiratory distress syndrome (ARDS) may help to investigate its role in recruitment and survival of airway neutrophils and other inflammatory or airway cells.

Previous studies showed a higher level of sOPN in asthma. Our study adds to these studies by showing that in asthma, the RTLF contains lower fraction of polymeric and higher fraction of monomeric and cleaved sOPN. Potential explanations for these differences are higher proteolytic cleavage and/or lower polymerization in airways of subjects with asthma. Polymerization of OPN is thought to be performed by TGM2, which a recent report has suggested to be increased in asthma [66]. As such, inhibition of TGM2 activity or higher proteolytic cleavage may be the more likely explanations for the presence of lower polymeric sOPN that we observed in asthma. Cleaved sOPN has been suggested to interact with CD44 [67], [68] and may play a role in the pathogenesis of asthma through this mechanism [69].

There are a few potential limitations to our study. First, we loaded equal volumes of BAL, and not equal amounts of total protein on our immunoblots. Our rationale is that all BAL procedures were performed by instilling the same amount (100 ml) of saline in subjects' right middle lobe, which should result in similar dilution of all subjects' RTLF. Loading different BAL volumes with the same amount of total protein could potentially obscure any real differences as the amount of total protein in airways is a function of airway biology and disease. In our analysis, we analyzed relative densitometry for BAL of each subject, which should bypass the potential confounding of equal volumes vs. equal protein amounts. In addition, we adjusted the measured absolute densitometries for total protein concentration by dividing the absolute densities by total protein concentration of each sample, and in our regression models, we included the total protein concentrations as a possible predictor. Second, sputum samples may be contaminated with saliva and may not exclusively represent the RTLF of lower airways. While our sputum induction method is different than the method that isolates and homogenizes mucus plugs as a representation of RTLF, it is the method adopted and in current use by the NIH Asthma and COPD Clinical Networks, and has been shown to be a valid method of RTLF assessment. In this method, saliva is cleared from the mouth before each expectoration of sputum and so there is minimal contamination by saliva. Furthermore, the quality of samples is assessed by measurement of squamous cells and those with high contamination with squamous cells are routinely excluded. The close correlation between BAL samples and induced sputum samples obtained by our induction method is reassuring in that this method likely provides a reasonable sample of RTLF. Third, our study is an observational study, and does not provide data on functional sequelae of polymerization or fragmentation of sOPN. While, a few studies have shown important functional consequences for polymerization of OPN [27], [32], further studies are needed to better understand the role of sOPN in lung pathophysiology. However, our study provides important information that will be useful in appropriate design of such studies particularly as it implies that any administered OPN may become polymerized *in vitro* or *in vivo* through the action of TGM2.

### Conclusions

In conclusion, our results suggest (1) that sOPN in human airways undergoes extensive post-translational modification by polymerization and proteolytic fragmentation, (2) that sOPN is more fragmented and less polymerized in subjects with mild to moderate asthma, and (3) that sOPN may contribute to recruitment or survival of alveolar macrophages.

## Supporting Information

Methods S1 **Description of lung function measurements, sputum induction, bronchoscopy, and sputum and BAL samples processing procedures.**
(DOC)

Table S1 **Characteristics of subjects in BAL group.** Values are expressed as mean ± SD. BMI: body mass index; PC_20_: provocative concentration of methacholine resulting in a 20% decrease in FEV_1_ compared with baseline. P-values are for comparisons (Student *t*-test) of concentrations of the variable between healthy and asthmatic subjects. Significant p-values are shown in bold.
(DOC)

Table S2 **Characteristics of subjects in sputum group.** Values are expressed as mean ± SD. BMI: body mass index; PC_20_: provocative concentration of methacholine resulting in a 20% decrease in FEV_1_ compared with baseline. P-values are for comparisons (Student *t*-test) of concentrations of the variable between healthy and asthmatic subjects. Significant p-values are shown in bold.
(DOC)

Table S3 **Concentrations of inflammatory cells and total protein in BAL.** Values are expressed as mean ± SD. P-values are for comparisons (Student *t*-test) of concentrations of the variable between healthy and asthmatic subjects. Significant p-values are shown in bold.
(DOC)

Table S4 **Concentrations of inflammatory cells and total protein in sputum.** Values are expressed as mean ± SD. P-values are for comparisons (Student *t*-test) of concentrations of the variable between healthy and asthmatic subjects. Significant p-values are shown in bold.
(DOC)
