# Supplementary material for: Secreted Osteopontin Is Highly Polymerized in Human Airways and Fragmented in Asthmatic Airway Secretions
Source: PLoS One. 2011 Oct 21;6(10):e25678. doi: 10.1371/journal.pone.0025678 (PMC3198733; doi:10.1371/journal.pone.0025678)
Supplement: Methods S1 — Description of lung function measurements, sputum induction, bronchoscopy, and sputum and BAL samples processing procedures. (DOC) [file pone.0025678.s001.doc]

**Methods S1**

*Lung function measurements.* Spirometry was done before and 15 min after administration of 180 μg of albuterol by metered dose inhaler using a Collins Survey dry rolling-seal spirometer (Warren E. Collins Co., Braintree, MA) and according to American Thoracic Society performance criteria [1].

*Methacholine challenge*. Airway responsiveness was determined by the FEV_1_ response to the inhalation of nebulized phosphate-buffered saline (PBS) solution followed by doubling concentrations (0.25, 0.5, 1.0, 2.0, 4.0, 8.0. and 16.0 mg/ml) of methacholine in PBS solution delivered via a dosimeter (DeVilbiss No.646; Somerset, PA) at the rate of 0.01 ml per breath following a protocol modified from the Lung Health Study [2]. PC_20_ compared to the post-PBS baseline was calculated by log-linear interpolation.

*Induced sputum procedures.* The sputum induction procedure of our laboratory is a modification of the NIH Asthma Clinical Research Network procedure [3,4] and has been described previously [5]. Briefly, subjects were pretreated with 240 μg of albuterol, and spirometry was performed before and 15 min after the administration of albuterol to ensure that the post-bronchodilator FEV_1_ was >60% of the predicted value for each subject. Subjects then underwent sputum induction in an isolation booth to control for any possible airborne infections. Sputum induction was performed by the inhalation of nebulized 3% sterile saline solution for 20 min. At each 2-min interval, subjects were asked to clear saliva from their mouth by spitting into a sterile plastic container and then cough up sputum into a second such container. A 20-min sputum induction time was used to obtain respiratory samples from peripheral airways and the distal lung [6]. All subjects tolerated 20 min of the sputum induction procedure without difficulty. For quality control, a sputum sample was considered to be inadequate if its volume was <1 ml or if its percentage of squamous cells was >80% [4]. The volume of the induced sputum sample was determined and an equal amount of 0.1% dithiothreitol was added. The sample was homogenized by gentle mixing and then was placed in a shaking water bath at 37°C for a minimum of 15 min.

*Bronchoscopy and lavage procedures.* Our laboratory's procedures of bronchoscopy and BAL have been previously published in detail [7]. Briefly, intravenous access was established, supplemental oxygen was delivered, and the upper airways were anesthetized with topical lidocaine. Sedation with intravenous midazolam and fentanyl was used as needed for subject comfort. The bronchoscope was introduced through the mouth and vocal cords into the airways. The bronchoscope was then directed into the right middle lobe where lavage was performed with two 50-ml aliquots of 0.9% saline warmed to 37°C. After bronchoscopy, each subject was observed for an approximate 2-hour recovery period. Recovered lavage fluid was immediately placed on ice.

*Total and differential cell counts.* Total cells were counted on unspun aliquots of BAL or sputum using a hemocytometer. Differential cell counts were obtained from slides prepared using a cytocentrifuge (25 g for 5 min) and were stained with Diff-Quik (Dade Behring, Düdingen, Switzerland). Two hundred cells were counted independently by each of two blinded individuals, and the mean of these counts was used in the data analysis.

*Sputum and BAL fluid processing.* The sputum and BAL fluids were then centrifuged at 180 *g* for 15 minutes, and the supernatant was separated and recentrifuged at 1,400 *g* for 15 minutes to remove any cellular debris before freezing at -80°C.

*Total protein measurement.* Total protein in induced sputum and BAL was measured using a bicinchoninic acid (BCA) protein assay (Pierce Protein Research Product, Thermo Fisher Scientific Inc., Rockford, IL).

References

1. (1995) Standardization of Spirometry, 1994 Update. American Thoracic Society. Am J Respir Crit Care Med 152: 1107-1136.

2. Kanner RE, Connett JE, Altose MD, Buist AS, Lee WW, et al. (1994) Gender difference in airway hyperresponsiveness in smokers with mild COPD. The Lung Health Study. Am J Respir Crit Care Med 150: 956-961.

3. Gershman NH, Wong HH, Liu JT, Mahlmeister MJ, Fahy JV (1996) Comparison of two methods of collecting induced sputum in asthmatic subjects. Eur Respir J 9: 2448-2453.

4. Fahy JV, Boushey HA, Lazarus SC, Mauger EA, Cherniack RM, et al. (2001) Safety and reproducibility of sputum induction in asthmatic subjects in a multicenter study. Am J Respir Crit Care Med 163: 1470-1475.

5. Criqui GI, Solomon C, Welch BS, Ferrando RE, Boushey HA, et al. (2000) Effects of azithromycin on ozone-induced airway neutrophilia and cytokine release. Eur Respir J 15: 856-862.

6. Gershman NH, Liu H, Wong HH, Liu JT, Fahy JV (1999) Fractional analysis of sequential induced sputum samples during sputum induction: evidence that different lung compartments are sampled at different time points. J Allergy Clin Immunol 104: 322-328.

7. Arjomandi M, Witten A, Abbritti E, Reintjes K, Schmidlin I, et al. (2005) Repeated exposure to ozone increases alveolar macrophage recruitment into asthmatic airways. Am J Respir Crit Care Med 172: 427-432.
