# Supplementary material for: Secreted Osteopontin Is Highly Polymerized in Human Airways and Fragmented in Asthmatic Airway Secretions
Source: PLoS One. 2011 Oct 21;6(10):e25678. doi: 10.1371/journal.pone.0025678 (PMC3198733; doi:10.1371/journal.pone.0025678)
Supplement: Table S1 — Characteristics of subjects in BAL group. Values are expressed as mean ± SD. BMI: body mass index; PC20: provocative concentration of methacholine resulting in a 20% decrease in FEV1 compared with baseline. P-values are for comparisons (Student t-test) of concentrations of the variable between healthy and asthmatic subjects. Significant p-values are shown in bold. (DOC) [file pone.0025678.s002.doc]

| **Subjects in BAL group** | **Non-asthmatic subjects (N=12)** | **Asthmatic subjects (N=21)** | **p-value** |
| --- | --- | --- | --- |
| **Age (years)** | 31.8 ± 6.0 | 35.4 ± 9.7 | 0.25 |
| **Sex (female)** | 5 (41.7 %) | 8 (38.1 %) | 0.85 |
| **Height (cm)** | 168.0 ± 10.6 | 168.2 ± 8.6 | 0.96 |
| **Weight (kg)** | 70.3 ± 13.6 | 78.7 ± 22.8 | 0.25 |
| **BMI (kg/m^2^)** | 24.8 ± 3.2 | 27.8 ± 7.9 | 0.21 |
| **FEV_1_ (L)** | 3.78 ± 0.72 | 2.92 ± 0.69 | **0.02** |
| **FEV_1_ (% predicted)** | 104.1 ± 14.0 | 76.4 ± 12.3 | **0.0004** |
| **FVC (L)** | 4.60 ± 1.0 | 4.52 ± 1.1 | 0.86 |
| **FVC (% predicted)** | 104.8 ± 13.5 | 96.1 ± 10.6 | 0.0.16 |
| **FEV_1_/FVC** | 0.83 ± 0.04 | 0.66 ± 0.37 | **0.0001** |
| **PC_20_ (mg/ml)** | > 8.0 | 1.97 ± 4.2 | - |
| **Atopy** | 8 (66.7%) | 21 (100%) | **0.004** |
