# Supplementary material for: Secreted Osteopontin Is Highly Polymerized in Human Airways and Fragmented in Asthmatic Airway Secretions
Source: PLoS One. 2011 Oct 21;6(10):e25678. doi: 10.1371/journal.pone.0025678 (PMC3198733; doi:10.1371/journal.pone.0025678)
Supplement: Table S2 — Characteristics of subjects in sputum group. Values are expressed as mean ± SD. BMI: body mass index; PC20: provocative concentration of methacholine resulting in a 20% decrease in FEV1 compared with baseline. P-values are for comparisons (Student t-test) of concentrations of the variable between healthy and asthmatic subjects. Significant p-values are shown in bold. (DOC) [file pone.0025678.s003.doc]

| **Subjects in Sputum group** | **Non-asthmatic subjects (N=27)** | **Asthmatic subjects (N=20)** | **p-value** |
| --- | --- | --- | --- |
| **Age (years)** | 35.2 ± 9.1 | 33.0 ± 8.0 | 0.415 |
| **Sex (female)** | 14 (58.3 %) | 12 (63.2 %) | 0.457 |
| **Height (cm)** | 170.0 ± 8.0 | 170.4 ± 10.1 | 0.894 |
| **Weight (kg)** | 66.3 ± 10.5 | 70.2 ± 12.1 | 0.323 |
| **BMI (kg/m^2^)** | 22.7 ± 2.6 | 24.4 ± 3.7 | 0.125 |
| **FEV_1_ (L)** | 3.48 ± 0.58 | 3.24 ± 0.86 | 0.312 |
| **FEV_1_ (% predicted)** | 94.8 ± 13.7 | 89.1 ± 16.5 | 0.249 |
| **FVC (L)** | 4.07 ± 0.79 | 4.00 ± 0.78 | 0.796 |
| **FVC (%predicted)** | 93.1 ± 16.8 | 93.0 ± 13.1 | 0.982 |
| **FEV_1_/FVC** | 0.85 ± 0.11 | 0.79 ± 0.07 | 0.061 |
| **PC_20_ (mg/ml)** | > 8.0 | 2.59 ± 2.60 | - |
| **Atopy** | 14 (51.9%) | 16 (84.2%) | **0.023** |
