# Supplementary material for: Secreted Osteopontin Is Highly Polymerized in Human Airways and Fragmented in Asthmatic Airway Secretions
Source: PLoS One. 2011 Oct 21;6(10):e25678. doi: 10.1371/journal.pone.0025678 (PMC3198733; doi:10.1371/journal.pone.0025678)
Supplement: Table S3 — Concentrations of inflammatory cells and total protein in BAL. Values are expressed as mean ± SD. P-values are for comparisons (Student t-test) of concentrations of the variable between healthy and asthmatic subjects. Significant p-values are shown in bold. (DOC) [file pone.0025678.s004.doc]

| **BAL** | **All subjects (N=33)** | **Non-asthmatic Subjects (N=12)** | **Asthmatic Subjects (N=21)** | **p-value** |
| --- | --- | --- | --- | --- |
| **Total Leukocyte (x10^4^ cells/ml)** | 18.0 ± 6.4 | 20.6 ± 7.4 | 16.8 ± 5.6 | 0.126 |
| **Macrophage (%)** | 86.2 ± 7.4 | 84.2 ± 6.8 | 87.4 ± 7.7 | 0.231 |
| **Neutrophil (%)** | 3.2 ± 3.1 | 3.0 ± 2.6 | 3.2 ± 3.3 | 0.824 |
| **Lymphocyte (%)** | 10.0 ± 7.6 | 12.6 ± 5.8 | 8.5 ± 8.2 | 0.141 |
| **Eosinophil (%)** | 0.6 ± 1.1 | 0.3 ± 0.4 | 0.8 ± 1.3 | 0.171 |
| **Total Protein (µg/ml)** | 88.9 ± 33.2 | 81.0 ± 37.4 | 93.5 ± 30.6 | 0.31 |
