# Supplementary material for: Secreted Osteopontin Is Highly Polymerized in Human Airways and Fragmented in Asthmatic Airway Secretions
Source: PLoS One. 2011 Oct 21;6(10):e25678. doi: 10.1371/journal.pone.0025678 (PMC3198733; doi:10.1371/journal.pone.0025678)
Supplement: Table S4 — Concentrations of inflammatory cells and total protein in sputum. Values are expressed as mean ± SD. P-values are for comparisons (Student t-test) of concentrations of the variable between healthy and asthmatic subjects. Significant p-values are shown in bold. (DOC) [file pone.0025678.s005.doc]

| **Sputum** | **All subjects (N=47)** | **Non-asthmatic Subjects (N=27)** | **Asthmatic Subjects (N=20)** | **p-value** |
| --- | --- | --- | --- | --- |
| **Total Leukocyte (x10^4^ cells/ml)** | 80.6 ± 62.0 | 88.1 ± 73.5 | 70.0 ± 40.3 | 0.336 |
| **Macrophage (%)** | 42.5 ± 13.5 | 41.7 ± 13.6 | 43.5 ± 13.7 | 0.667 |
| **Neutrophil (%)** | 52.8 ± 14.0 | 53.5 ± 14.8 | 51.8 ± 13.2 | 0.706 |
| **Lymphocyte (%)** | 3.7 ± 1.9 | 4.3 ± 1.9 | 2.9 ± 1.4 | **0.011** |
| **Eosinophil (%)** | 1.0 ± 1.8 | 0.4 ± 0.7 | 1.7 ± 2.4 | **0.020** |
| **Total Protein (mg/ml)** | 2.0 ± 0.6 | 2.2 ± 0.7 | 1.6 ± 0.4 | **0.005** |
